# Amplification of Signal on Cell Surfaces in Molecular Cascades

**DOI:** 10.3390/cells12242858

**Published:** 2023-12-18

**Authors:** Sergei Rudchenko, Steven Taylor, Nenad Milosavic, Maria Rudchenko, Betina Wedderhoff Tissi, Markus Y. Mapara, Milan N. Stojanovic

**Affiliations:** 1 Division of Experimental Therapeutics, Department of Medicine, Columbia University, 630W 168th St., Box 84, New York, NY 10032, USA; 2 Hunter College, City University of New York, 695 Park Avenue, New York, NY 10065, USA; 3 Columbia Center for Translational Immunology, Department of Medicine, Columbia University, 630W 168th St., Box 84, New York, NY 10032, USA; 4 Department of Biomedical Engineering, Columbia University, 630W 168th St., Box 84, New York, NY 10032, USA

**Keywords:** nanotechnology, molecular programming, DNA strand displacement cascades, flow cytometry

## Abstract

We can formulate mixtures of oligonucleotide–antibody conjugates to act as molecular cascade-based automata that analyze pairs of cell surface markers (CD markers) on individual cells in a manner consistent with the implementation of Boolean logic—for example, by producing a fluorescent label only if two markers are present. While traditional methods to characterize cells are based on transducing signals from individual cell surface markers, these cascades can be used to combine into a single signal the presence of two or even more CDs. In our original design, oligonucleotide components irreversibly flowed from one antibody to another, driven by increased hybridizations, leading to the magnitude of the final signal on each cell being determined by the surface marker that was the least abundant. This is a significant limitation to the precise labeling of narrow subpopulations, and, in order to overcome it, we changed our design to accomplish signal amplification to a more abundant cell surface marker. We show the AMPLIFY function on two examples: (1) we amplify the fluorescent label from the CD19 marker onto a fivefold more abundant CD45, and (2) we amplify broadly distributed CD45RA to a more constant marker, CD3. We expect this new function to enable the increasingly complex Boolean analysis of cell surfaces.

## 1. Introduction

The simplest way to make molecules compute [1,2,3] and behave as automata [4,5], or, arguably, as robots [6,7,8], is through a sense–compute–actuate paradigm. Accordingly, we often state that a collection of molecules computes when it interacts with multiple other molecular species as inputs and follows a truth table to implement an analytical, diagnostic, or therapeutic action based on these inputs.

A branch of molecular computing and robotics focuses on interactions of molecules with a cell to perform an analysis of its surface markers [6,8,9,10]. The traditional targeting of cells is based on the selective recognition of a single marker [11,12,13] that is a characteristic of the targeted cell population. In contrast, in molecular computing, multiple cell surface moieties are analyzed by molecules, without any human input, before a single label is produced. It is very rare for a single cell surface marker to be restricted only to a single targeted cell; therefore, molecular computing approaches are often pursued to advance the therapeutic goal of minimizing side effects on cells that share the primary target of therapeutic antibodies but diverge in a secondary target. The goals and concepts of this subfield are, thus, shared with conditional approaches to bifunctional antibodies [14,15] or the synthetic biology engineering of CAR-T therapies that target two markers [16]. Therefore, the selective in vivo targeting of cells using a combinatorial cascade approach of surface markers that are not expressed together on normal tissues should increase the therapeutic index and prevent on-target-off-tumor effects. Another clinical application for molecular cascade reactions lies in the field of cell graft engineering. The precise ex vivo selection or depletion of a target cell population may be of benefit in several clinical contexts. Thus, the depletion of naïve T cells as defined by the expression of CD45RA without eliminating non-T cells expressing CD45RA (e.g., B cells and NK cells) may allow the prevention of Graft-versus-Host Disease (GvHD) [17] while otherwise retaining the diverse cellular graft composition and immune reconstitution.

A fundamental limitation of our initial design was its stoichiometric nature; namely, these cascades were construed in a way that made the lowest-abundance surface marker control the extent of labeling. For example, in our previous paper [8], CD20 was expressed on the cell surface in about two-fold lower abundance than CD45. Therefore, CD20 controls the magnitude of the signal produced in both DNA-based digital logic circuits [18] (in our case, a cascade reaction) in the YESCD20YESCD45 and YESCD45YESCD20 directions. Through the current work, we address this issue by engineering automata (or cascade reactions) to implement an AMPLIFY function [19] on the cell surface. 

## 2. Results and Discussion

### 2.1. Design Considerations for Amplification Cascade Reaction

**Figure 1:**
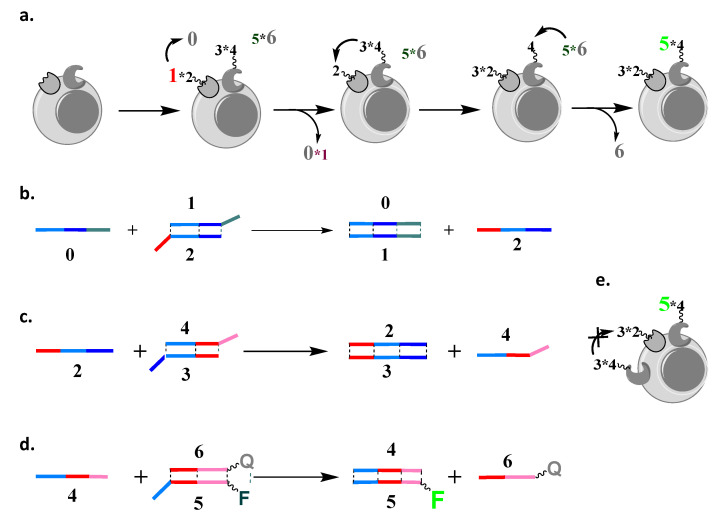
Stoichiometric cascade [18] design. (**a**) A stoichiometric cascade that analyzes two cell surface markers consists of four elements. The progress of the cascade can be monitored using fluorescence. Elements 1∗2 and 3∗4, which are both partial DNA duplexes, are delivered to two cell surface markers through antibodies to which **2** and **4** are covalently conjugated, while **0** (single stranded DNA) and 5∗6 (partial DNA duplex) are in solution; the addition of **0** triggers the cascade. (**b**–**d**) Individual steps of the strand displacement cascade, with the products of one strand reaction (e.g., **2** and **4**) initiating the next steps in the cascade, with the final step triggering the acquisition of a fluorescent signal. (**e**) The cascade stops after a single assessment of the cell surface, being unable to label another molecule of the second cell surface marker, which is here present in an excess of the first.

Consider two cell surface markers present on the same cell in proximity, which allows physical contact between oligonucleotides conjugated to antibodies directed to these markers (Figure 1a). Our goal is to achieve the transfer of an oligonucleotide element from one marker to the other. If this transfer generates a label for flow cytometry, then only cells that have both markers in proximity will be labeled. This is a problem that is solved also by proximity ligation assays [20]. However, here, a permanent crosslink is formed between moieties that target each marker, which we need to avoid while cells are alive, due to possible side effects.

In our previous design [8], we implemented a stoichiometric cascade in which the transfer of oligonucleotides was irreversible (Figure 1). The overall reaction on the cell surface was as follows:

0+1∗2+3∗4+5∗6→0∗1+2∗3+4∗5+6,

where **0** is a single-stranded oligonucleotide that triggers the start of the cascade reaction. Oligonucleotide **2** is attached to an antibody and forms a partial DNA duplex with oligonucleotide **1**. Likewise, oligonucleotide **4** is attached to an antibody and forms a partial DNA duplex with oligonucleotide **3**. Oligonucleotide **2** can also form a duplex with **3** if **1** is removed, and, likewise, **4** can also form a duplex with oligonucleotide **5** if **3** is removed. Colored fonts represent fluorescently labeled oligonucleotides: in red, with Cy5; in green, with fluorescein; in black, fluorochromes are either quenched or not attached; strands 0 and **6** are labeled with quenchers Iowa Black RQ for Cy5 and Iowa Black FQ for fluorescein, respectively.

The first step was the addition of a single-stranded trigger oligonucleotide, **0** (Figure 1b), initiating strand displacement and the transfer of **1** to it from its complex with **2**, per

0+1∗2→0∗1+2

(with **2** being covalently attached to the first antibody).

In this design, all oligonucleotides have three domains [8,21]; for example, **0** consists of three domains and is fully complementary to oligonucleotide **1**, which consists of three antisense domains (matched with color, Figure 1a). In contrast, **2** has only two complementary domains to **1** (light blue and dark blue); thus, when **0** encounters the complex between **1** and **2** (or 1∗2), there is an irreversible transfer of **1** to **0**. Note that **2** is now not in a complex and, thus, ready for the next step in the cascade (Figure 1c), in which it will accept **3**. This next step is triggered by a newly revealed dark blue domain, which is uniquely positioned next to the light blue domain in only one oligonucleotide, **2**:

(1) 2+3∗4→2∗3+4

(with **4** attached to the second antibody and triggering another step, Figure 1d).

A weakness of this design is pictorialized in Figure 1e; namely, after one execution, all elements that participate in the cascade will become irresponsive. Therefore, if there is one surface marker that is limited in its abundance, the signal on the other marker will be capped by it.

**Figure 2:**
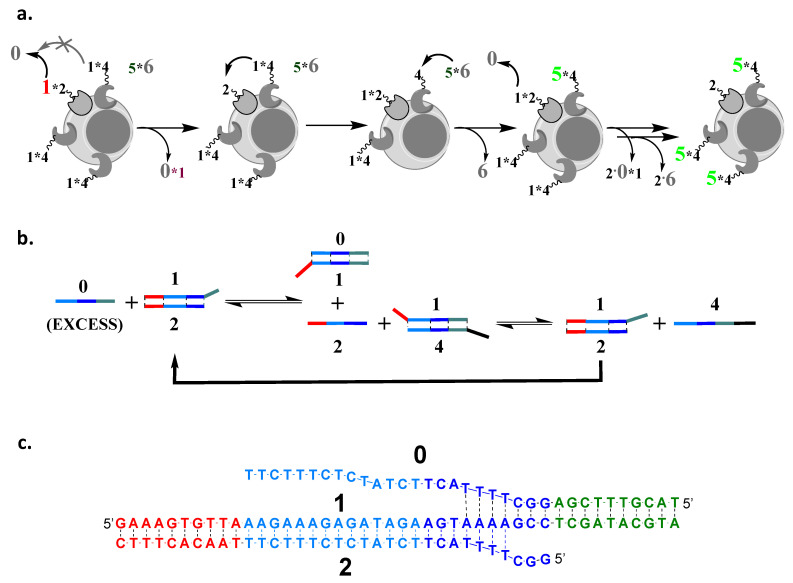
Design of cascades with amplification. (**a**) Amplification requires that we regenerate free **2** and that **1** can be transferred to **0** only from **2**, but not from **4**, which leads to one **2** being able to catalyze the formation of multiple free **4** on the cell surface. We can fluorescently label individual components, depending on what we want to observe; here, for example, we labeled **1** only in the initial complex with **2**. The final product of the first two steps, single-stranded oligonucleotide **4**, can then irreversibly interact with 5∗6 (cf. Figure 3a). (**b**) Sequences are designed using a four-domain design and reversible interactions, until the last step. (**c**) An example of the cascade sequences used in the first strand-transfer step—domains are shown in different colors. The full cascade is given in Figure 3 and Supplementary Materials.

In order to address this weakness to allow a multiple assessment while sharing components, and, thus, amplify the signal beyond the level of the limiting cell surface marker, we need to somehow regenerate free **2** from the product 3∗2 as it is formed (Figure 1). For this purpose, we can make **1** and **3** identical, i.e., we can use 1∗4 to target the second cell surface marker, instead of 3∗4. Furthermore, **4** and **2** need to have different domains within them complementary to **1**, in order to avoid a direct reaction of 1∗4 with **0** (the overhang domain of **1** in 1∗2 is not available in 1∗4). Overall, then, if, in proximity to 1∗2 on the cell surface, there are n elements of 1∗4, the cascade reaction with n-fold amplification will have the form below (cf. Figure 2a):

$$\begin{array}{l} k\cdot 0+1*2+n\cdot 1*4+l\cdot 5*6\\ \;\;\;\;\;\;\;\;\to 0*1+\left(k-1\right)\cdot 0+1*2+\left(n-1\right)\cdot 1*4+4*5+\left(l-1\right)\cdot 5*6+6\to \cdots \\ \;\;\;\;\;\;\;\;\to \left(k-n\right)\cdot 0+n\cdot 0*1+2+n\cdot 4*5+\left(l-n\right)\cdot 5*6+n\cdot 6 \end{array}$$

(where k and l ≫n, with 2 and 4 attached to antibodies immobilized on the cell surface).

We accomplished this requirement by using four instead of three domain sequences, relying, for the first time, on expanding concepts from equilibrating seesaw gates [19,22] (Figure 2). Here, in the first step, both **0** and **2** each have three different domains complementary with **1**, each leading to the equilibrium shuffling of **1** between them (Figure 2b). Further, this equilibrium is coupled to another one, in which **1** is shuffled between **2** and **4**, with the resulting single-stranded **4** able to participate in an irreversible reaction with 5∗6 (cf. Figure 1d). 

The cascade is driven towards products by an excess of **0** and using the irreversible strand displacement element to cap the cascade only in the last step. The full set of sequences, including the description of the mismatches that were introduced to increase the rate of cascade response, are shown in the Supplementary Materials; here, we limit ourselves to showing the sequences of components that participate in the first step. The cascade was first optimized in the solution phase (Supplementary Materials), and then we moved to demonstrate it on cell surfaces. 

### 2.2. AMPLIFY(YESCD19YESCD45) Cascade Reaction

**Figure 3:**
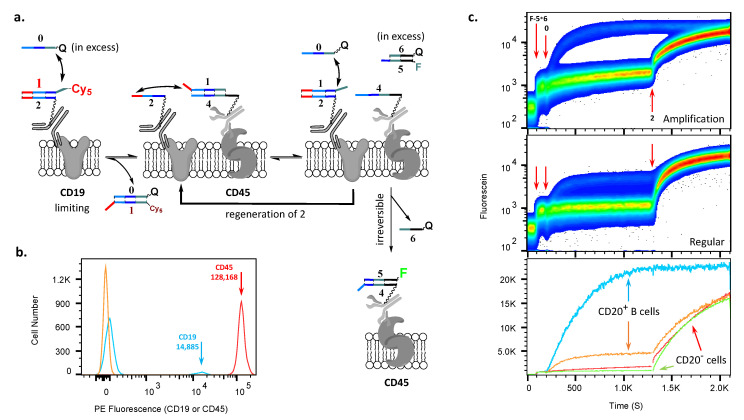
Implementation of the AMPLIFY(YESCD19YESCD45) cascade on PBMCs. (**a**) Schematics of the amplification cascade operation on the cell surface. (**b**) Histogram comparing amounts of CD19 and CD45 per cell. (**c**) Upper panel shows the time course of the acquisition of fluorescein in the amplification cascade—namely, the reaction of **4** with F-5∗6-FQ in solution. The middle panel shows the analogous stoichiometric cascade, i.e., without amplification (see Supplementary Materials). Red arrows show events, in order—additions of F-5∗6-FQ, **0**-RQ, and, finally, **2**. The lower panel shows an overlay of results from the upper and middle channels in mean fluorescence intensity, demonstrating the amplification (blue trace) of the fluorescein signal relative to the stoichiometric cascade (orange trace) on the cell surface of B cells (CD19^pos^CD20^pos^) and CD20^neg^ cells (red and green traces). Note: upper and middle panel ordinates are log scales, and lower panel ordinate is a linear scale.

We selected AMPLIFY(YESCD19YESCD45), per Figure 3a, for the first demonstration on human peripheral blood mononuclear cells (PBMCs). These two markers, CD19 and total CD45, are reportedly [23,24] present at about 20,000 and 200,000 copies on the surfaces of human B cells, with other types of lymphocytes lacking CD19. In our hands, with phycoerythrin (PE) conjugates of the same antibody clones that will be used in the cascade, we determined that the ratio between the expression of these two molecules on the surfaces of B lymphocytes was 8.8 (Figure 3b and Figure S4e). This ratio of expression made it possible for us to study the increase in the accumulation of fluorescein on the surfaces of B cells during the amplification cascade reaction and to make comparisons with the stoichiometric cascade reaction.

In order to demonstrate amplification (Figure 2bandFigure 3a), we first applied conjugates of anti-CD19 and anti-CD45 antibodies with duplexes Cy5-1∗2 (**1** was labeled here with Cy5) and 1∗4 (**1** was unlabeled), respectively. The ratio of oligonucleotide-to-antibody for CD19 was about 1.9 and for CD45 was about 1.2. Further, to confirm the subpopulation at which the cascade occurs, we also additionally labeled B cells within PBMCs with anti-CD20 antibodies conjugated with Pacific Blue fluorochrome. Once the conjugates had been deposited, we added fluorescein-labeled 5∗6 complex (F-5∗6-FQ) to the solution and then initiated the cascade with **0** (Figure 3c, addition events are shown by red arrows). 

The individual steps in the amplification were as follows (Figure 3a). First, Cy5-**1** is transferred to **0**-RQ (which is observed by flow cytometry) from **2** (on CD19), allowing **2** to acquire **1** back from proximal **4** (on CD45; this transfer step is not monitored in flow), regenerating 1∗2 on CD19, although without Cy5. This transfer releases **4** and enables it to acquire, in an irreversible step, F-**5** from its solution-phase complex with **6**-FQ (monitored in panel 3c). Multiple turnovers are enabled because newly generated, unlabeled 1∗2 reenters the cascade through another reaction with an excess of **0**. 

For comparison, on the same PBMC samples, we also performed a stoichiometric YESCD19YESCD45 cascade (i.e., without amplification), where oligonucleotide **1** was replaced with strand **3** in the complex of 1∗4 conjugated to CD45 antibodies (please see Methods for details) (Figure 3a, direct comparison in Figure S4). Viewed together in the fluorescein channel, in which we monitored the acquisition of F-**5**, these two experiments, one with and one without amplification, established that there were multiple turnovers occurring on the cell surfaces. Further, the observed signal was about 5.5-fold lower for the stoichiometric cascade than with the amplification one (Figure 3c, lower panel, cf. blue and orange traces at saturation), i.e., we could estimate that, on the cell surface, for each molecule of antibody against CD19, about 5–6 molecules of antibodies against CD45 markers acquired fluorescein from the solution. 

At the end of each experiment, both with and without amplification, we further added free single-stranded **2** in excess to remove any **1** from the unreacted complex 1∗4 attached to CD45 surface markers, which freed all **4** to acquire F-**5**, allowing us to obtain the maximum possible signal and assess the extent of amplification. Because there was no further increase observed in reactions that implemented amplification cascades, these experiments revealed that all available CD45-targeting conjugates on cells reacted during amplification. 

The cascade reactions reached their saturation in both cases. Since the ratio of oligonucleotide-to-protein for the CD19 antibody is approximately 1.6 times higher than the same ratio for the CD45 antibody, this explains the difference between the ratio of expression between CD45 and CD19 molecules and the efficiency of accumulation of fluorescein on the cell surface during regular and amplification cascade reactions.

### 2.3. AMPLIFY(YESCD45RAYESCD3) Cascade Reaction

**Figure 4:**
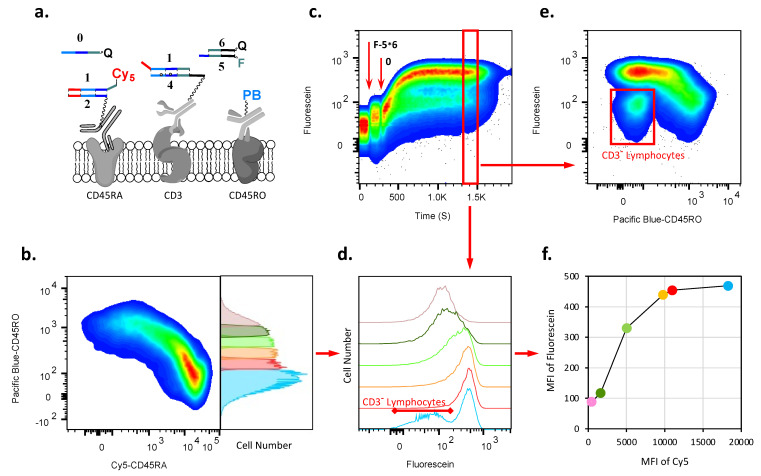
Demonstration of AMPLIFY(YESCD45RAYESCD3) cascade on lymphocyte surfaces. (**a**) Components of the cascade before triggering it. (**b**) CD45RA and CD45RO, two isoforms of CD45, are expressed differently in number on CD3^pos^ T cell subsets. We show the distribution of CD45RA acquired via anti-CD45RA conjugated with Cy5-1∗2 vs. CD45RO expression on lymphocyte surfaces before the cascade is triggered by the addition of **0**-RQ strand. The pseudocolor density plot shows that as the amount of CD45RO increases on the cell surface, the amount of CD45RA decreases in a linear manner. The right panel shows the frequency distribution of lymphocytes based on the expression level of CD45RO. We arbitrarily separated cells into six fractions, which were individually monitored (Figure S7b). The first fraction (in blue) also contained all subpopulations of lymphocytes without or with very low expression of CD45RO and, consequently, with a high level of expression of CD45RA, including non-T cells (CD3^neg^), while the last fraction (in pink) expressed almost no CD45RA. (**c**) Flow cytometry monitoring of the acquisition of F-**5** on the surfaces of lymphocytes. Time course of the cascade reaction on CD3^pos^ T cells: fluorescein-labeled **5** is taken up from solution by **4** on the surfaces of CD3^pos^ T cells after the addition of trigger **0**-RQ, resulting in the separation of CD3^pos^ T cells from CD3^neg^ lymphocytes. (**d**) Frequency distributions of acquired F-**5** as the result of cascade vs. CD45RO expression on lymphocyte surfaces based on six fractions from (**b**) (colors match as in b). Histograms present cells gated on the time course between 1300 and 1500 s for each fraction of CD45RO in (**b**). (**e**) The pseudocolor density plot presents lymphocytes gated on the time course between 1300 and 1500 s, i.e., when the reaction reaches saturation. This density plot shows the fluorescein accumulation vs. amount of CD45RO on the cell surface. Red, orange, and yellow are areas of high cell density. The vertical distribution of the red–yellow spot reflects cells with similar amounts of fluorescein accumulated on the cell surface via the amplification cascade, while the amount of CD45RO increases, and, consequently, the amount of CD45RA decreases. (**f**) Result of the accumulation of fluorescein vs. amount of CD45RA on the surfaces of T cells presented as a **m**ode of histograms of **f**luorescence **i**ntensity (MFI) in (**d**). The amount of CD45RA is presented as the MFI of the initial distributions of Cy5 in fractions within (**b**).

The different expression of isoforms of CD45, the leukocyte common antigen, is associated with T cell development both within the thymus and among peripheral blood and is used for the characterization of T lymphocyte subsets. The two isoforms, CD45RA and CD45RO, are summatively expressed at nearly constant numbers on the lymphocytes of healthy individuals. The CD45RA isoform is present on the surfaces of all populations of lymphocytes: T cells, B cells, and innate lymphoid cells, including NK cells. For our second demonstration, we chose to amplify the presence of different amounts of CD45RA on the cell surfaces of different subsets of T lymphocytes from peripheral blood. Thus, here, the challenge was to amplify different quantities of this marker to similar levels of final fluorescence. As for the other surface marker, the one to which we amplified CD45RA, we chose CD3, a T cell marker present at steady levels. Thus, we implemented an AMPLIFY(YESCD45RAYESCD3) cascade, as shown in its initial state in Figure 4a.

In order to follow the amplification by flow cytometry, we used a gating strategy based on the expression of CD45RO, the second major isoform of CD45. Thus, we could use an anti-CD45RO antibody as an independent surrogate (on which the signal does not change during cascade reactions) to quantify by proxy the CD45RA amount on the cell surfaces before (Figure 4b) and during cascade execution (Figure S7); here, the higher the signal of CD45RO is, the less of CD45RA there is on the cell, and together they provide the total CD45. Accordingly, in Figure 4b, we show the distribution of cells labeled with an anti-CD45RA-targeting antibody conjugate with Cy5-1∗2, a reagent used in the first step of the cascade, and an anti-CD45RO-targeting antibody conjugated with Pacific Blue fluorophore (PB). 

The principles behind this cascade are the same as in the previous example, except that the antibodies are changed (for a technical demonstration of amplification in solution and on beads, see Figures S5 and S6). Once the cascade is triggered, we cannot monitor Cy5, which is removed; therefore, to follow the relative distributions of CD45RA, we use fluorescently labeled CD45RO instead. After the cascade’s initiation, the result of the cascade (which must go through CD45RA) is monitored by the accumulation of fluorescein-labeled strand **5** by its surface-bound (via anti-CD3) complement **4** (Figure 4c). When the cascade reaction reaches saturation (Figure 4d,e), we observe that the fluorescein levels accumulate to almost the same amount on the cell surfaces vs. the increasing amount of CD45RO (and, respectively, decreasing expression of CD45RA) on a two-dimensional pseudocolor density plot(Figure 4e and Figure S7c). Together with a technical demonstration that amplification is possible in solution (Figure S5) and on beads (see Figure S6), this is a convincing argument for amplification occurring.

To obtain more fine-grained insights into the levels of amplification, we extracted the cascade results for the individual fractions of T cells, as defined by the levels of CD45RO (and, consequently, CD45RA). We split these cells into six separate fractions (Figure 4b and Figure S7a) showing the first and the last steps of the cascade (removal of Cy5-**1** and acquisition of F-**5**). We can assess the relative initial levels of Cy5-**1**, which vary, and the relative final levels of F-**5**, demonstrating up to threefold amplification (Figure 4f and Figure S7b, see numbers). The first fraction, which had the lowest levels of CD45RO, contained also CD3^neg^ cells (e.g., B cells and innate lymphoid cells), which were separated as a distinct subpopulation that, per design, escaped cascade-based labeling with F-**5** (Figure 4e and Figure S7b,c). The sixth fraction, which contained cells displaying little or no CD45RA molecules, had an increase in fluorescence that was similar to the background that we had observed in previous cascades on CD19^neg^ cells (Figure 3c).

## 3. Conclusions

Our results unambiguously establish that the cascade design results in the amplification of signals. While solution-phase cascades can amplify the initial signal by up to 100-fold, amplification cascades on cell surfaces are limited in the number of recycling steps by their environment. The cascade can now be introduced as a part of larger circuits to extend the number of steps that can be executed within automata. These studies should pave the way for clinical applications in the context of cellular therapy—for example, to eliminate naïve CD45RA^pos^ T cells for the prevention of GvHD in the setting of allogeneic transplant, or to select certain cell populations like CD62L^pos^CD45RA^pos^ naïve/stem cells or memory T cells for genetic manipulation (i.e., expression of chimeric antigen receptor) in the context of immune effector cell therapy to prevent cytokine release syndrome.

## 4. Materials and Methods

### 4.1. Materials

The Pierce Immobilized Reductant Column 2 mL (Thermo Scientific, Waltham, MA, USA, #77701) and Zeba Spin Desalting Column 0.5 mL 7K MWCO (Thermo Scientific, Waltham, MA, USA, #89882) were used. 

The oligonucleotides were (5′→3′)

**0** =/5IAbRQ/TACGTATCGAGGCTTTTACTTCTATCTCTTTCTT;
**1** = GAAAGTGTTAAAGAAAGAGATAGAAGTAAAAGCCTCGATACGTA;
**1**(Cy5) = GAAAGTGTTAAAGAAAGAGATAGAAGTAAAAGCCTCGATAC
GTA/3Cy5Sp/;
**2** =/5ThioMC6-D/GGCTTTTACTTCTATCTCTTTCTTTAACACTTTCCTATACTT
ATGCACTT;
**3** = GAAAGTGTTAAAGAAAGAGATTGAAGTAAATGCCTC;
**4** =/5ThioMC6-D/CTTTACGATTTGGTTACGTATCGAGGCATTTACTTCAATCT
CTTTCTT;
**5** =/5BioTinTEG/AGTAAATGCCTCGATACGTAACCAAATCGTTAAGCC/36-FAM/;
**6** =/5IABkFQ/GGCTTAACGATTTGGTTACGTATCGA.

Abbreviations: 5IAbRQ is a dark quencher (500–700 nm) modification; 5ThioMC6-D is a thiol (disulfide form) modification; 3Cy3Sp is a Cy3 fluorophore modification; 5BioTinTEG is a biotin-TEG modification; 36-FAM is a fluorescein fluorophore modification; 5IABkFQ is a dark quencher (420–620 nm) modification.

All oligonucleotides were designed to have minimal secondary structures and were commercially manufactured by Integrated DNA Technologies Inc. (Coralville, IA, USA) (see Figure S1a).

**Antibodies:**

All antibodies were acquired from BioLegend Inc. (San Diego, CA, USA). Purified anti-CD3 (clone HIT3a), anti-CD19 (clone HIB19), anti-CD45 (clone HI30), and anti-CD45RA (clone HI100) for conjugation with oligonucleotides were purchased in concentrations of at least 2 mg/mL. The same clones of anti-CD19 and anti-CD45 antibodies conjugated with phycoerythrin (PE) and with known ratios of the number of PE molecules per molecule of antibody (1.30 for CD19, Cat. No. 302208, Lot B342131, and 1.27 for CD45, Cat. No. 304008, Lot B358564) were used for the determination of the number of molecules (CD19 and CD45) expressed on the cell surface. Pacific Blue conjugated anti-CD20 (clone 2H7, Cat. No. 302328) and anti-CD45RO (clone UCHL1, Cat. No. 304216) antibodies were used for the selection of B cells (CD20) and T cells with different expression of CD45RA (CD45RO) by flow cytometry.

### 4.2. Methods

**Maleimide functionalization of sulfhydryl-oligonucleotide** (see Figure S1b):

*Part A—Activation of disulfide-protecting oligonucleotide to give the sulfhydryl:* A 1 mL solution of 50 nmoles of disulfide-oligonucleotide in activation buffer (100 mM sodium phosphate, pH 8.0, with 1 mM EDTA, 5 mM DTT) was prepared. An Immobilized Reductant Column was activated with 10 mL of the activation buffer, followed by washing with 5 mL of 100 mM sodium phosphate, pH 8.0, 1 mM EDTA buffer. The column was loaded with the oligonucleotide solution, followed by capping and standing for 1 h on the bench. The column was then washed with 10 mL of PBS, pH 6.8, 5 mM EDTA buffer (PBSE). The activated oligonucleotide was isolated from the column by applying 10 mL of PBS, pH 6.8, 5 mM EDTA buffer containing a high salt concentration (1 M NaCl). The product eluted mostly in the third mL. Note—for the third and fourth mL, it is best to apply the elution buffer in 250 µLaliquots and then determine the concentrations of the fractions by absorption on 260 nm, and to combine fractions that contain oligonucleotide.

*Part B—Addition of the maleimide functional group:* 100 equivalents of BMH were dissolved in an equal volume—relative to the volume of the oligonucleotide from the above steps—of DMSO. The resulting BMH/DMSO solution was rapidly pipetted and mixed into the sulfhydryl-oligonucleotide solution and then incubated at room temperature for 1 h. The reaction mixture was then divided into approximately 350 µL aliquots and 1.5 mL of ice-cold absolute ethanol added to each. The mixtures were left to stand at −20 °C for 45 min and then centrifuged at high speed for 5 min to pellet the precipitate. The supernatant was removed and the pellets placed in a desiccator overnight to dry.

**Disulfide reduction of IgG and conjugation** (see Figure S1b):

*Disulfide reduction of IgG*: IgG (1 mg in 2 × 100 µL, 6.67 nmoles) was buffer exchanged into TRIS buffer (0.1 M, pH 8.0) using two 0.5 mL Zeba desalting columns (100 µL of IgG solution per column). DTT (100 mM stock in the TRIS buffer—made by dissolving 15 mg in 1 mL) was then added to the buffer-exchanged IgG to give a final concentration of 5 mM DTT. The reaction mixture was incubated at 37 °C for 30 min. DTT was then removed using two sequential 0.5 mL Zeba desalting columns equilibrated with phosphate buffer (PBS with 5 mM EDTA, pH 6.8), to ensure removal of the excess DTT.

*Conjugation of reduced IgG to maleimide-oligonucleotide:* Four equivalents of maleimide-functionalized oligonucleotide were combined (on ice) with one equivalent of the reduced IgG. The reaction mixture was incubated for 30 min on ice and then refrigerated at 4 °C overnight. A complementary strand was then added (1.5 equivalents, with respect to maleimide-oligonucleotide added previously) and the mixture incubated at room temperature for 30 min and then purified by gel filtration on an Äkta FPLC system refrigerated at 4 °C, with a Superdex 200 10-300GL column (GE Healthcare, Barrington, IL, USA), eluting with PBS buffer, pH 6.8 (see Figures S2 and S3). For the long-term preservation of all conjugates, glycerol (45%) was added and they were stored at −20 °C.

**PBMC purification:**

PBMCs were purified from a Buffy Coat, acquired from anonymous (deidentified) donors and purchased from New York Blood Center, on Ficoll-Paque gradient (Cytiva Sweden AB, Uppsala, Sweden) with SepMate-50 tubes (StemCell Technologies, Vancouver, BC, Canada), according to the manufacturer’s instructions.

**Flow cytometry methods and in situ preparation of the stoichiometric and amplification cascades:**

*Amplification cascade:*

1. PBMCs (25 × 10^6^/mL) were pre-incubated with anti-CD32 antibodies (12.5 ng/mL, Clone IV.3, STEMCELL Technologies, Vancouver, BC, Canada) and Shredded Salmon Sperm DNA (125 ug/mL, Ambion, Austin, TX, USA) in buffer (PBS supplemented with 0.5% (*w*/*v*) BSA and 2 mM EDTA) at room temperature for 10 min.
2. The conjugates of antibodies participated in the cascade reaction together with the corresponding Pacific Blue conjugated antibody were added to PBMCs in an amount that was determined after previous titration, to be sure that all antigens on the cell surface would be saturated with minimal non-specific binding registered. The mixture was incubated on ice for 30 min with periodic gentle mixing.
3. Samples were washed twice with up to 15 mL buffer by centrifugation (350 *g* × 10 min, at 5 °C).
4. The final pellet was resuspended to obtain a concentration of cells of 50 × 10^6^ PBMCs per ml of buffer at room temperature.
5. About 5 × 10^6^ PBMCs (100 µL) were resuspended in a final volume of 500 µL in a 5 mL tube for flow cytometry and the sample subjected to flow cytometry at a rate 12 µL/min. After the monitoring of the baseline during the first 100 s, the duplex F-**5∗6** was added to sample to reach a final concentration of about 700 nM; after the second 100 s, the strand **0** was added to reach a final concentration about 200 nM. In some experiments, after the next 1100 s, strand 2 was added to obtain a final concentration of about 400 nM.

*Stoichiometric cascade for YESCD19YESCD45:*

1. PBMCs (25 × 10^6^/mL) were pre-incubated with anti-CD32 antibodies (12.5 ng/mL, Clone IV.3, STEMCELL Technologies) and Shredded Salmon Sperm DNA (125 ug/mL, Ambion) in PBS supplemented with 0.5% (*w*/*v*) BSA and 2 mM EDTA (Buffer) at room temperature for 10 min.
2. The CD45-(**1∗4**) conjugate was added to PBMCs in an amount that was determined after previous titration, to be sure that all antigens on the cell surface would be saturated, with minimal non-specific binding registered. The mixture was incubated on ice for 30 min with periodic gentle mixing.
3. Samples were washed twice with up to 15 mL of buffer by centrifugation (350 *g* × 10 min, at 5 °C).
4. Cells were resuspended in a concentration of 25 × 10^6^/mL in buffer and strand 2 was added to obtain a final concentration 400 nM with subsequent incubation at room temperature for 30 min.
5. Samples were washed twice with up to 15 mL of buffer by centrifugation (350 *g* × 10 min, at 5 °C).
6. Cells were resuspended at a concentration of 25 × 10^6^/mL in buffer and strand 3 was added to obtain a final concentration of 400 nM with subsequent incubation on ice for 30 min.
7. Samples were washed twice with up to 15 mL buffer by centrifugation (350 *g* × 10 min, at 5 °C).
8. The CD19-(**1∗2**) conjugate together with Pacific Blue anti-CD20 antibodies was added to PBMCs in an amount that was determined after previous titration, to be sure that all antigens on the cell surface would be saturated with minimal non-specific binding registered. The mixture was incubated on ice for 30 min with periodic gentle mixing.
9. Samples were washed twice with up to 15 mL of buffer by centrifugation (350 *g* × 10 min, at 5 °C).
10. The final pellet was resuspended to obtain a concentration of cells at 50 × 10^6^ PBMCs per ml of buffer at room temperature.
11. About 5 × 10^6^ PBMCs (100 µL) were resuspended in a final volume 500 µL in a 5 mL tube for flow cytometry and the sample subjected to flow cytometry at a rate of 12 µL/min. After the monitoring of the baseline during the first 100 s, the duplex F-**5∗6** was added to the sample to obtain a final concentration of about 700 nM; after the second 100 s, the strand **0** was added to obtain a final concentration of about 200 nM. After the next 1100 s, strand 2 was added to obtain final concentration of about 400 nM.

All experiments were repeated at least twice with PBMCs from different healthy donors. For flow cytometry analyses of samples, a BD FACS Calibur with Cytek DxP upgrade, BD FACS Canto, and Cytek Athena was used. For data analysis, the FlowJo software (latest version v 10) was used. 

## Data Availability

Data are contained within the article and Supplementary Materials.

## Supplementary Materials

The Supplementary Materials contain all procedures, conjugate characterization, and Supplementary Figures S1–S7. The following supporting information can be downloaded at: https://www.mdpi.com/article/10.3390/cells12242858/s1.
