# Supplementary material for: Amplification of Signal on Cell Surfaces in Molecular Cascades
Source: Cells. 2023 Dec 18;12(24):2858. doi: 10.3390/cells12242858 (PMC10742280; doi:10.3390/cells12242858)
Supplement: Supplementary file 1 [file cells-12-02858-s001.zip › cells-2653233-supplementary.pdf]

Amplification of Signal on Cell Surfaces in Molecular Cascades

Sergei Rudchenko [1,*], Steven Taylor [1], Nenad Milosavic [1], Maria Rudchenko [1], Betina Wedderhoff Tissi [1,4], Markus Y. Mapara[3] and Milan N. Stojanovic [1,2,*]

1 Division of Experimental Therapeutics, Department of Medicine, Columbia University, 630W 168th St., Box 84, New York, New York 10032, United States

2 Hunter College, City University of New York, 695 Park Avenue, New York, NY 10065, USA

3 Columbia Center for Translational Immunology, Department of Medicine, Columbia University, 630W 168th St., Box 84, New York, New York 10032, United States

4 Department of Biomedical Engineering, Columbia University, 630W 168th St., Box 84, New York, NY 10032, USA

* Correspondence: sar2173@cumc.columbia.edu (S.R.); mns18@cumc.columbia.edu (M.N.S.)

Materials

Pierce Immobilized Reductant Column 2 mL (Thermo Scientific, 77701). Zeba Spin Desalting Column 0.5 mL 7K MWCO (Thermo Scientific, 89882).

The oligonucleotides are (5'→3'):

0 = /5IAbRQ/TACGTATCGAGGCTTTTACTTCTATCTCTTTCTT

1 = GAAAGTGTTAAAGAAAGAGATAGAAGTAAAAGCCTCGATACGTA

1(Cy5) = GAAAGTGTTAAAGAAAGAGATAGAAGTAAAAGCCTCGATACGTA/3Cy5Sp/

2 = /5ThioMC6-D/GGCTTTTACTTCTATCTCTTTCTTTAACACTTTCCTATACTTATGCACTT

3 = GAAAGTGTTAAAGAAAGAGATTGAAGTAAATGCCTC

4 = /5ThioMC6-D/CTTTACGATTTGGTTACGTATCGAGGCATTTACTTCAATCTCTTTCTT

5 = /5BioTinTEG/AGTAAATGCCTCGATACGTAACCAAATCGTTAAGCC/36-FAM/

6 = /5IABkFQ/GGCTTAACGATTTGGTTACGTATCGA

Abbreviations: 5IAbRQ is a dark quencher (500-700nm) modification; 5ThioMC6-D is a thiol (disulfide form) modification; 3Cy3Sp is a Cy3 fluorophore modification; 5BioTinTEG is a biotin-TEG modification; 36-FAM is a fluorescein fluorophore modification; 5IABkFQ is a dark quencher (420-620 nm) modification.

All oligonucleotides were designed to have minimal secondary structure and were commercially manufactured by Integrated DNA Technologies Inc. (Coralville, IA).

Antibodies:

All antibodies were acquired from BioLegend Inc. (San Diego, CA). Purified ant-CD3 (clone HIT3a), anti-CD19 (clone HIB19), anti-CD45 (clone HI30), anti-CD45RA (clone HI100) for conjugation with oligonucleotides were purchased in concentration at least 2 mg/ml. Same clones of anti-CD19 and anti-CD45 antibodies conjugated with phycoerythrin (PE) and with known ratio of number of PE molecules per molecule of antibody (1.30 for CD19, Cat.No. 302208, Lot B342131, and 1.27 for CD45, Cat.No. 304008, Lot B358564) were used for the determination of the number molecules (CD19 and CD45) expressed on the cell surface. Pacific Blue conjugated anti-CD20 (clone 2H7, Cat.No. 302328) and anti-CD45RO (clone UCHL1, Cat.No. 304216) antibodies were used for selection of B-cells (CD20) and T-cells with different expression of CD45RA (CD45RO) by flow cytometry.

Methods

Maleimide functionalization of sulfhydryl-oligonucleotide:

Part A – Activation of disulfide-protect oligonucleotide to give the sulfhydryl: A 1 mL solution of 50nmoles of disulfide-oligonucleotide in activation buffer (100 mM sodium phosphate, pH 8.0, with 1 mM EDTA, 5 mM DTT) was prepared. An Immobilized Reductant Column was activated with 10mL of the activation buffer followed by washing with 5 mL of 100 mM sodium phosphate, pH 8.0, 1 mM EDTA buffer. The column was loaded with the oligonucleotide solution, followed by capping and standing for 1 hour on the bench. The column was then washed with 10 mL of PBS, pH 6.8, 5 mM EDTA buffer (PBSE). The activated oligonucleotide was isolated from the column by applying 10 ml of PBS, pH 6.8, 5 mM EDTA buffer containing high salt concentration (1 M NaCl). The product elutes mostly in the third mL: Note - for the third and fourth mL, it is best to apply the elution buffer in 250 uL aliquots, then determine concentrations of the fractions by Abs260nm, and combine fractions that contain oligonucleotide.

Part B – Addition of the maleimide functional group: 100 equivalents of BMH was dissolved in an equal volume - relative to the volume of the oligonucleotide from the above steps - of DMSO. The resulting BMH/DMSO solution was rapidly pipetted and mixed into the sulfhydryl-oligonucleotide solution, then Incubated at room temperature for 1 hour. The reaction mixture was then divided into approximately 350 uL aliquots and 1.5 mL of ice-cold absolute ethanol added to each. The mixtures were stood at -20°C for 45 mins, then centrifuge at high speed for 5 minutes to pellet the precipitate. The supernatant was removed and the pellets placed in a desiccator overnight to dry.

Disulfide reduction of IgG and conjugation:

Disulfide reduction of IgG - IgG (1 mg in 2x100 uL, 6.67 nmoles) was buffer exchanged into TRIS buffer (0.1 M, pH 8.0) using two 0.5 mL Zeba desalting columns (100 uL of IgG solution per column). DTT (100 mM stock in the TRIS buffer – made by dissolving 15 mg in 1 mL) was then added to the buffer exchanged IgG to give a final concentration of 5 mM DTT. The reaction mixture was incubated at 37°C for 30mins. DTT was then removed using two sequential 0.5mL Zeba desalting columns equilibrated with phosphate buffer (PBS with 5 mM EDTA, pH 6.8), to ensure removal of the excess DTT.

Conjugation of reduced IgG to maleimide-oligonucleotide:

Four equivalents of maleimide-functionalized oligonucleotide was combined (on ice) with one equivalent of the reduced IgG: The reaction mixture was incubated for 30mins on ice then refrigerated at 4°C overnight. Complementary strand was then added (1.5 equivalents, with respect to maleimide-oligonucleotide added previously), and the mixture incubated at room temperature for 30mins then purified by gel filtration on a Äkta FPLC system refrigerated at 4°C, with a Superdex 200 10-300GL column (GE Healthcare), eluting with PBS buffer, pH 6.8. For long term preservation of all conjugates, glycerol (45%) was added and stored at -20°C.

PBMC purification:

PBMCs were purified from Buffy Coat, acquired from anonymous (deidentified) donors and purchased from New York Blood Center, on Ficoll-Paque gradient (Cytiva Sweden AB, Uppsala, Sweden) with SepMate-50 tubes (StemCell Technologies, Vancouver, BC, Canada) according to the manufacturers instructions.

Flow cytometry methods and *in situ* preparation of the stoichiometric and amplification cascades:

Amplification cascade:

1. PBMCs (25x10^6/ml) were pre-incubated with anti-CD32 antibodies (12.5 ng/ml, Clone IV.3, STEMCELL Technologies) and Shredded Salmon Sperm DNA (125 ug/ml, Ambion) in buffer (PBS supplemented with 0.5% (w/v) BSA and 2 mM EDTA) at room temperature for 10 minutes.
2. The conjugates of antibodies participated in cascade reaction together with corresponding Pacific Blue conjugated antibody were added to PBMCs in an amount that was determined after previous titration to be sure that all antigens on cell surface will be saturated and minimal non-specific binding register. The mixture was incubated on ice for 30 minutes with periodical gentle mixing.
3. Samples were washed twice with up to 15 ml buffer by centrifugation (350gx10 min, at 5C).
4. The final pellet was resuspended to get a concentration of cells of 50x10^6 PBMCs per ml of buffer at room temperature.
5. About 5x10^6 PBMCs (100 ul) was resuspended in a final volume 500 ul in a 5 ml tube for flow cytometry and sample run by flow cytometry at a rate 12 ul/min. After monitoring of the baseline during the first 100 seconds, the duplex F-5*6 was added to sample to get final concentration of about 700 nM and after the second 100 seconds the strand 0 was added to get a final concentration about 200 nM. In some experiments, after the next 1100 seconds strand 2 was added to get a final concentration of about 400 nM.

Stoichiometric cascade for yesCD19yesCD45:

1. PBMCs (25x10^6/ml) were pre-incubated with anti-CD32 antibodies (12.5 ng/ml, Clone IV.3, STEMCELL Technologies) and Shredded Salmon Sperm DNA (125 ug/ml, Ambion) in PBS supplemented with 0.5% (w/v) BSA and 2 mM EDTA (Buffer) at room temperature for 10 minutes.
2. The CD45-(1*4) conjugate was added to PBMCs in an amount that was determined after previous titration to be sure that all antigens on cell surface will be saturated and minimal non-specific binding register. The mixture was incubated on ice for 30 minutes with periodical gentle mixing.
3. Samples were washed twice with up to 15 ml of buffer by centrifugation (350gx10 min, at 5C).
4. Cells were resuspended in concentration 25x10^6/ml in buffer and strand 2 was added to get a final concentration 400 nM with a following incubation at room temperature for 30 minutes.
5. Samples were washed twice with up to 15 ml of buffer by centrifugation (350gx10 min, at 5C).
6. Cells were resuspended at a concentration of 25x10^6/ml in Buffer and strand 3 was added to get final concentration 400 nM with a following incubation on ice for 30 minutes.
7. Samples were washed twice with up to 15 ml buffer by centrifugation (350gx10 min, at 5C).
8. The CD19-(1*2) conjugate together with Pacific Blue anti-CD20 antibodies was added to PBMCs in an amount was determined after previous titration to be sure that all antigens on the cell surface will be saturated and minimal non-specific binding register. The mixture was incubated on ice for 30 minutes with periodical gentle mixing.
9. Samples were washed twice with up to 15 ml of buffer by centrifugation (350gx10 min, at 5C).
10. The final pellet was resuspended to get a concentration of cells at 50x10^6 PBMCs per ml of buffer at room temperature.
11. About 5x10^6 PBMCs (100 ul) was resuspended in a final volume 500 ul in a 5 ml tube for flow cytometry and the sample run by flow cytometry at a rate of 12 ul/min. After monitoring of the baseline during the first 100 seconds the duplex F-5*6 was added to the sample to get a final concentration of about 700 nM and after the second 100 seconds the strand 0 was added to get a final concentration about 200 nM. In some experiments after the next 1100 seconds strand 2 was added to get final concentration of about 400 nM.

All experiments were repeated at least twice with PBMCs from different healthy donors. For flow cytometry analyses of samples, a BD FACS Calibur with Cytek DxP upgrade, BD FACS Canto, and Cytek Athena was used. For data analysis, FlowJo Software was used.

a)

```
       a          b          c          d          e          f          g

3'-GCAACTAGACTTCACGTATTCATATCCTTTCACAAT..........................................reg(0)
5'-CGTTGATCTGAAGTGCATAAGTATAGGAAAGTGTTA..........................................reg(1)
                                     3'-TTCTTTCTCTATCTTCATTTTCGGAGCTATGCAT.......ampl(0)
                           5'-GAAAGTGTTAAAGAAAGAGATAGAAGTAAAAGCCTCGATACGTA............(1)
           3'-TTCACGTATTCATATCCTTTCACAATTTCTTTCTCTATCTTCATTTTCGG......................(2)
                           5'-GAAAGTGTTAAAGAAAGAGATTGAAGTAAATGCCTC...................reg(3)
                                     3'-TTCTTTCTCTAACTTCATTTACGGAGCTATGCATTGGTTTAGCATTTC...(4)
                                                   5'-AGTAAATGCCTCGATACGTAACCAAATCGTTAAGCC.(5)
                                                             3'-AGCTATGCATTGGTTTAGCAATTCGG.(6)
```

b)

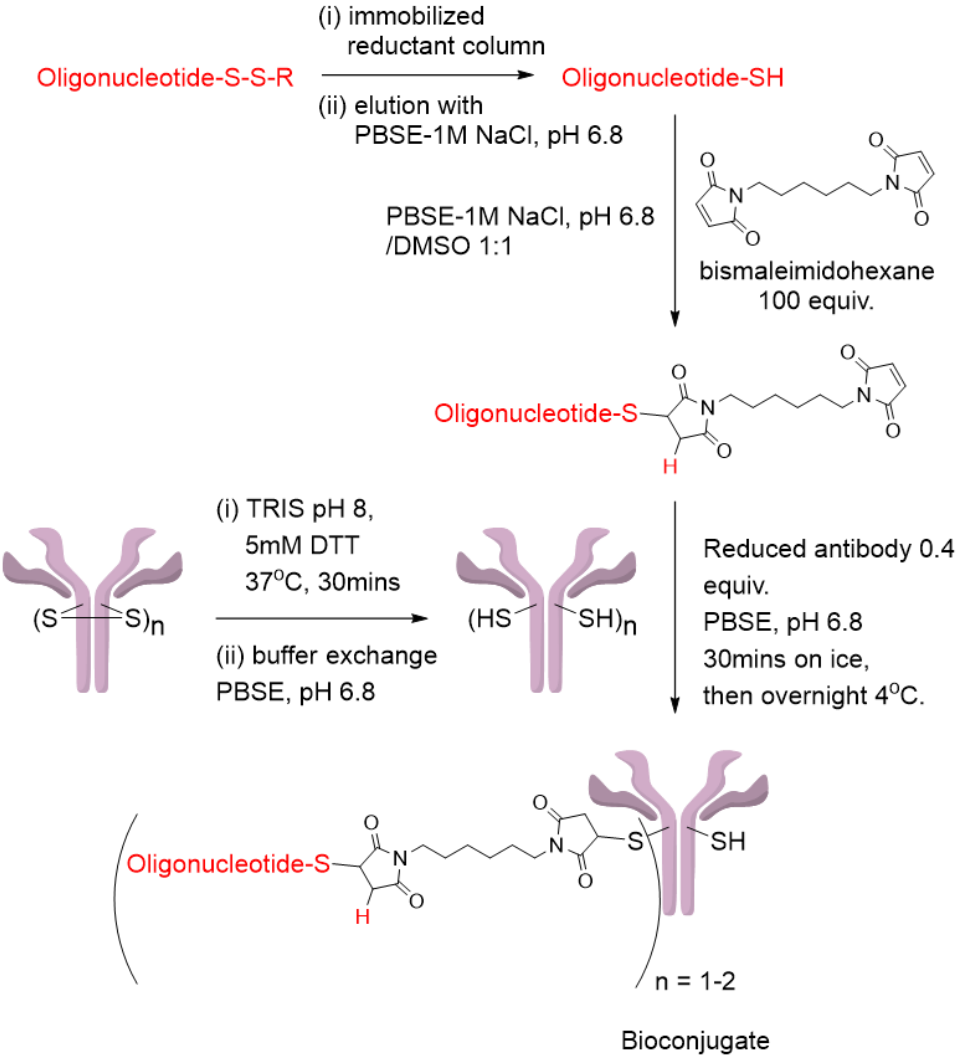

Figure S1. a) Oligonucleotide sequences and base parings (including mismatches) used in the amplification cascade and stoichiometric cascade. Oligonucleotides labeled ‘amp1’ or ‘reg’ are specifically for the amplification cascade, and stoichiometric cascade, respectively. Those not labelled are common to both cascades. **b**) Synthesis scheme for amplification cascade bioconjugates. For stoichiometric cascade biocojugates, 0.25 equivalents of reduced antibody was used in the last step.

a)

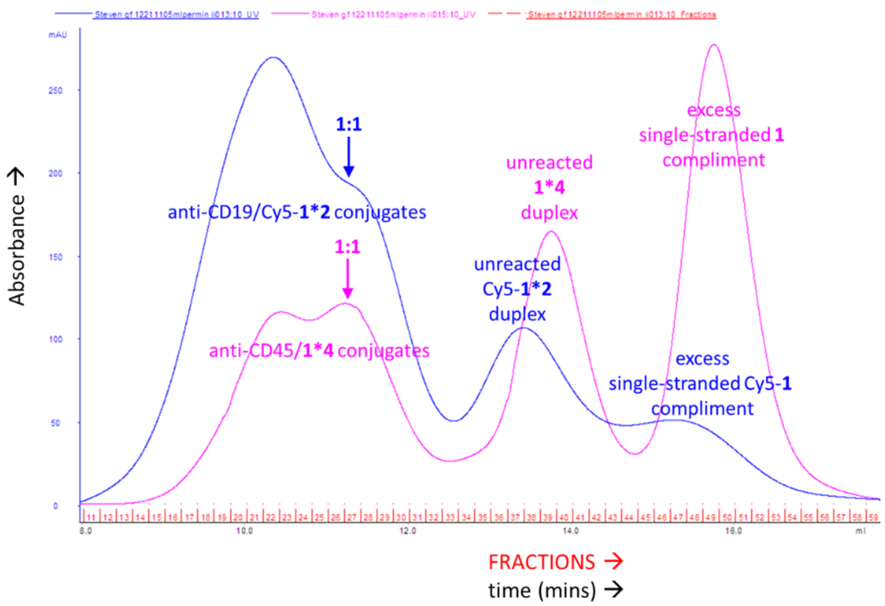

b)

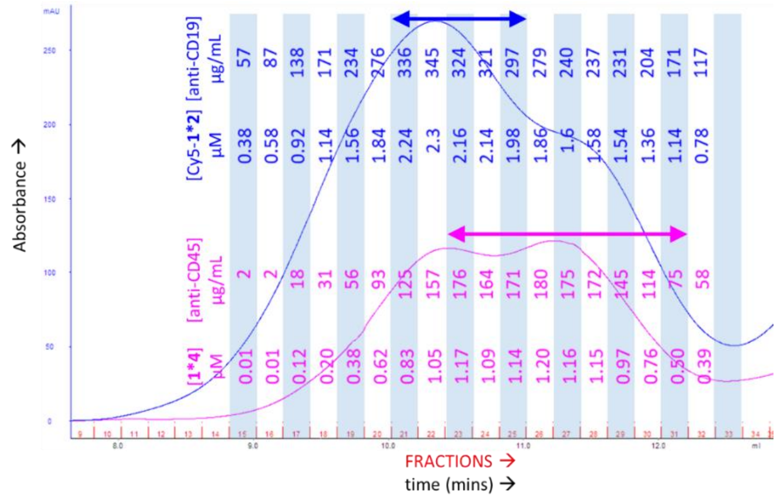

Figure S2. a) Äkta FPLC-size exclusion chromatography traces showing purification of anti-CD19- (blue) and antiCD45-(crimson) -oligonucleotide conjugate products after the reaction of disulfide-reduced antibody with 2.5 equivalents of maleimide-functionalized oligonucleotide followed by addition of oligonucleotide compliment (Cy5-**1** or **1**). Arrows indicate the assigned 1:1 ratio antibody:oligonucleotide-duplex peak. Expanded view of the conjugate products region with estimated calculated concentrations for fractions collected. All calculations were based on a 1:1 conjugates – however, based on a 2.5:1 reaction mixture, earlier eluting species are likely 2:1 conjugates, in which case concentrations give for the antibody concentration would be halved. Chromatography conditions, were not able to resolve the conjugate species. Based on preliminary analysis of each fraction by flow cytometry the fractions 21-25 (blue arrows) for anti-CD19 and fractions 23-31 (crimson arrows) for anti-CD45 antibodies were combined and after titration were used in future experiments. Our estimation of

the ratio of the number of duplex (Cy5-1*2) per molecule of anti-CD19 antibody is about 1.9 and the ratio of the number of duplex (1*4) per molecule of anti-CD45 antibody is about 1.2.

a)

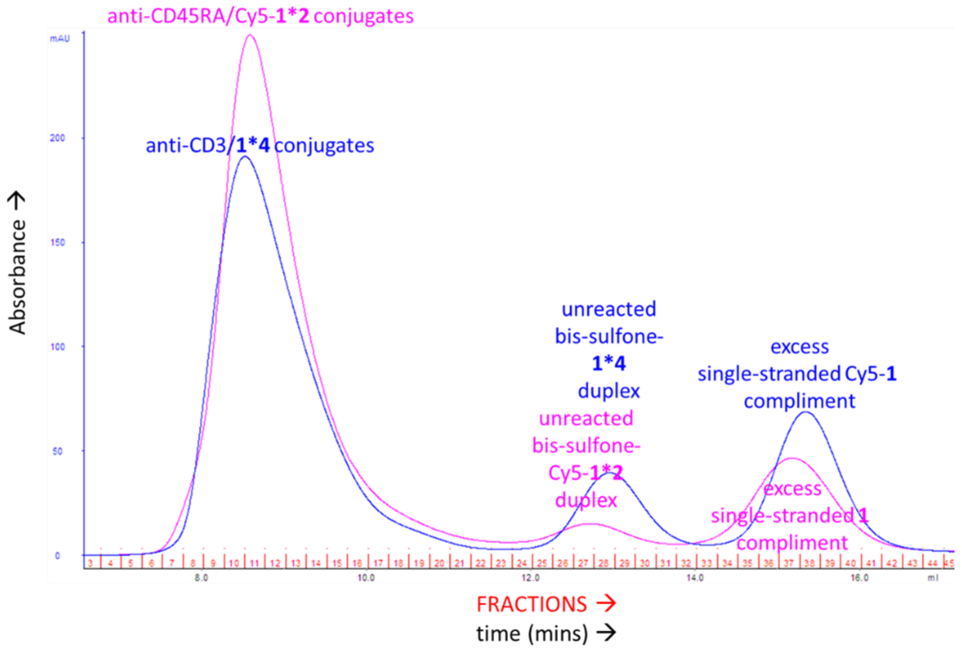

b)

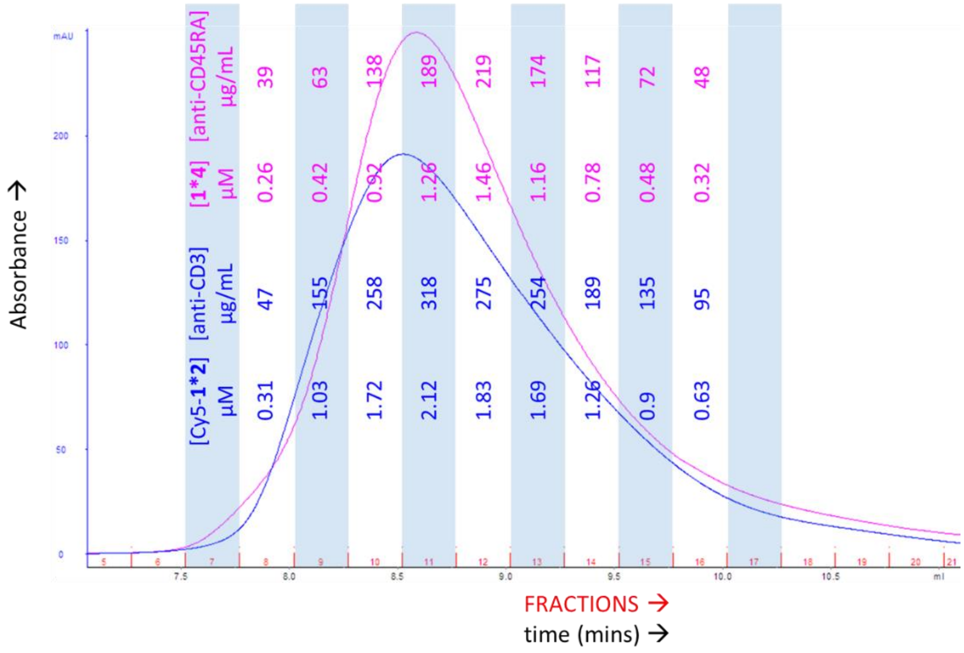

Figure S3. a) Äkta FPLC-size exclusion chromatography traces showing purification of anti-CD3- (blue) and antiCD45RA-(crimson) -oligonucleotide conjugate products after the reaction of disulfide-reduced antibody with maleimide-functionalized oligonucleotide followed by addition of oligonucleotide compliment (**1** or Cy5-**1**). All concentration calculations were based on a 1:1 conjugates. Chromatography conditions, were not able to resolve the conjugate species.

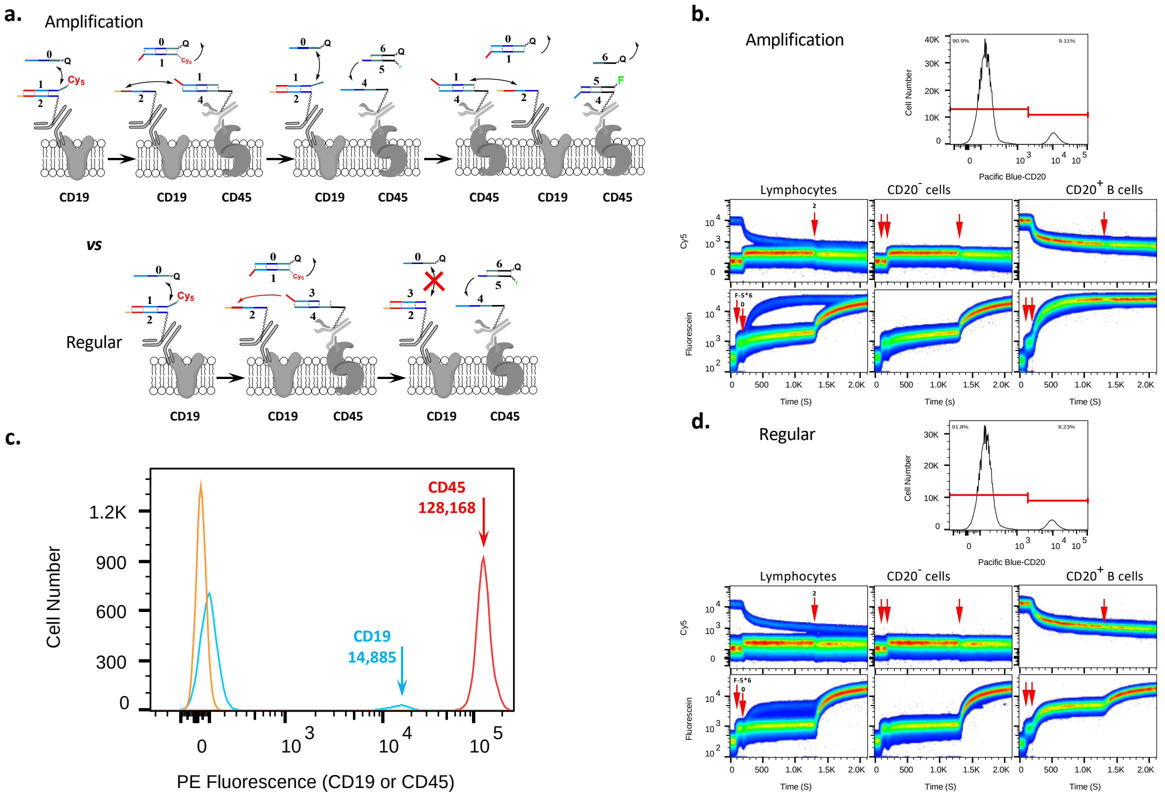

Figure S4. Comparison of the amplification YESCD19YESCD45 cascade and stoichiometric YESCD19YESCD45 cascade reactions. (a) Schematics of amplification *vs* stoichiometric cascades on the cell surface. (b) and (d) The time courses of acquisition of fluorescein (from solution F-**5*6**-Q) on strand **4** on the cell surface in the amplification (b) and stoichiometric (d) cascade reactions on $CD20^{pos}$ B-cells and $CD20^{neg}$ lymphocytes. (c) Histogram comparing amounts of CD19 and CD45 per cell. Knowing the efficiency of conjugation of anti-CD19 and anti-CD45 antibodies with PE (please, see Material and Methods) from these data, we are able estimate the ratio between expression of CD45 and CD19:

$$\frac{128{,}168}{1.27} \times \frac{1.3}{14{,}885} \cong 8.8.$$

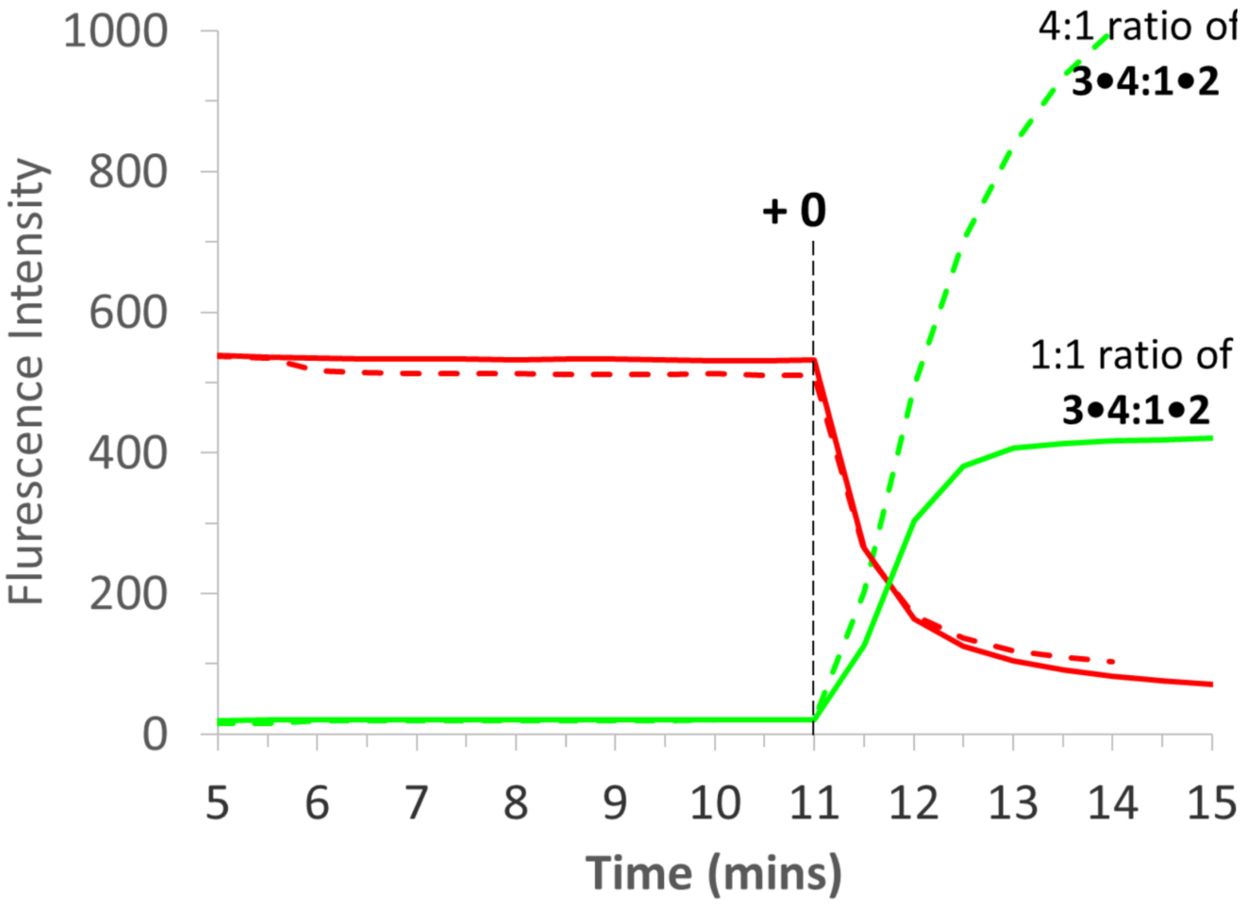

Figure S5 - Solution phase testing of anti-CD45RA-**2***Cy5-**1** and anti-CD3-**4***1 amplification conjugates in PBS buffer. All components except **0** were added prior to T = 5mins. Red traces are Cy5 channel and green traces are fluorescein channel. Dotted lines have 4x higher concentration of α-CD45RA conjugate than α-CD3 conjugate. Solid lines have both conjugates at 1x amounts.

a.

Preparation of Samples		
Antibodies	Sample A	Sample B
CD45RA-(Cy5-1▯2-PB)	0.05 µM	0.01 µM
CD45RA	-	0.04 µM
CD3-(1'▯4)	0.05 µM	0.05 µM

b.

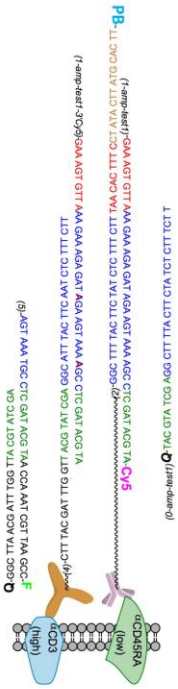

d.

$$\frac{1279-369}{4101-344} = 0.24$$

$$\frac{1822-31}{1906-32} = 0.956$$

c.

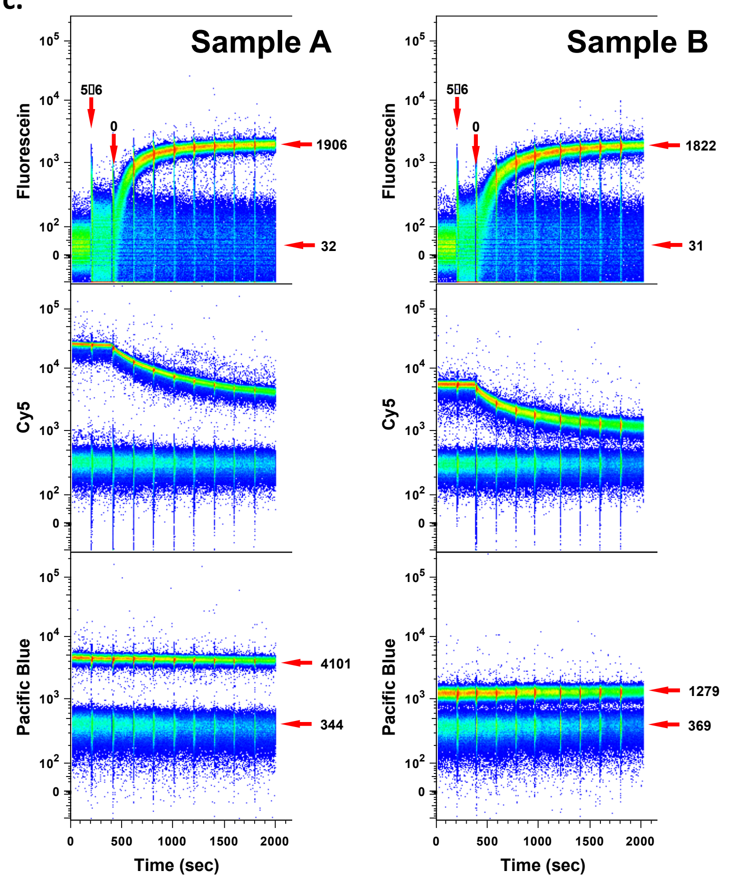

Figure S6. Demonstration of AMPLIFY(YESCD45RAYESCD3) cascade reaction on beads. BD CompBeads anti-mouse Ig, kappa (BD Biosciences, Cat.No. 552843) were used to capture CD45RA-(Cy-**1*2**-PB) and CD3-(**1*4**) conjugates (b) through Ig kappa light chain according the manufacturers instructions. (a) Two samples (A and B) were prepared with different concentrations of CD45RA-(Cy5-**1*2**-PB) conjugate. Pacific Blue attached directly to strand 2 was used to calculate the relative amount of CD45RA-(Cy5-**1*2**-PB) conjugate in both samples. (c) Time courses of acquisition of fluorescein on strand **4** on beads from F-**5*6**-Q in solution (upper panels); middle panels – monitoring the removal of Cy5-**1** on CD45RA conjugate on beads after triggering the cascade reaction with **0**; lower panels demonstrate stable levels of CD45RA-(**2**-PB) on beads during the cascade reaction. (d) Samples A and B, with a difference of about four times the amount of conjugate CD45RA-(Cy-**1*2**-PB) on beads, show about the same level of accumulation of F-**5**, thus demonstrating amplification in Sample B compare with Sample A.

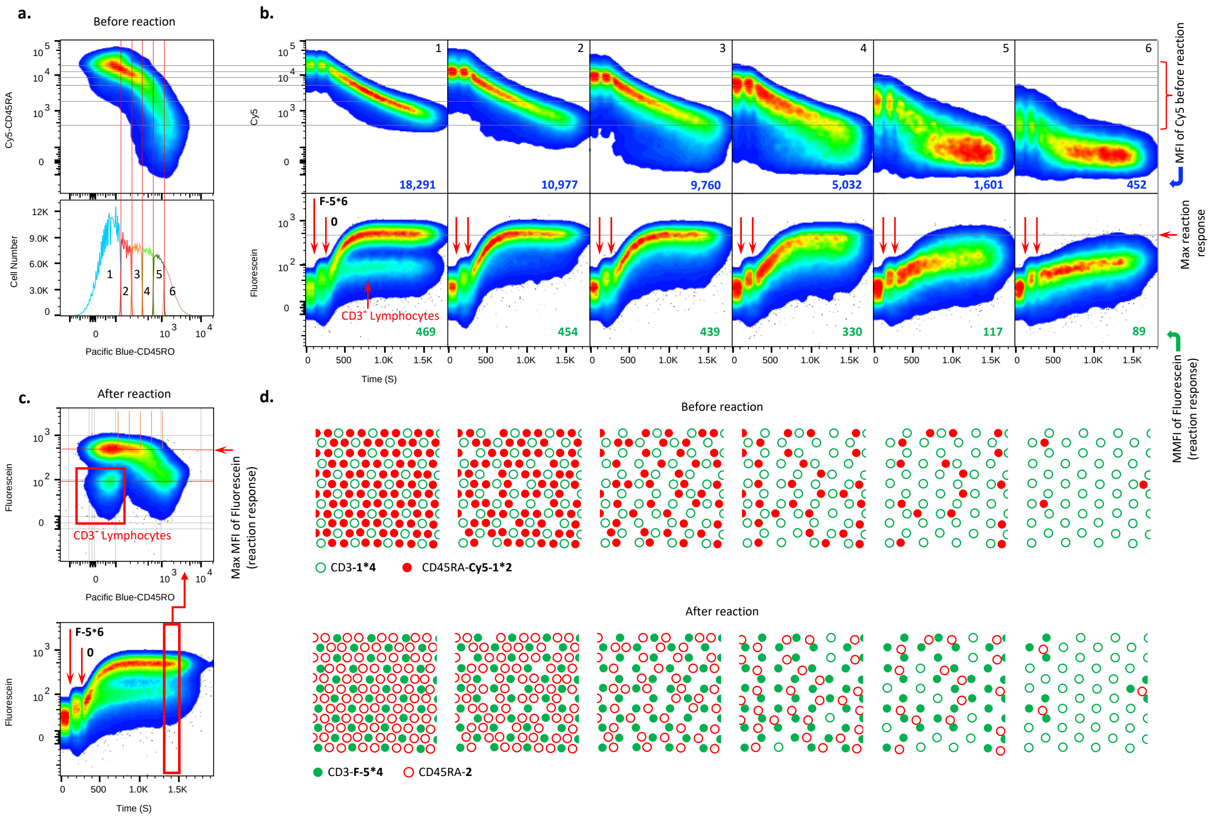

Figure S7. Demonstration of yesCD45RAyesCD3 amplification cascade on lymphocytes surfaces. (a) Gating strategy to show efficiency of cascade reaction based on different expression of CD45RO. CD45RA and CD45RO, two isoforms of CD45 differently expressed on CD3+ T-cell subsets. CD45 expression is fairly constant in normal individuals[21]. Distribution of CD45RA conjugated with **Cy5-1*2** duplex is presented before initiation of cascade reaction. (b) and (d) Present results of the amplification cascade reaction monitoring on CD3+ T-cell subsets with different expression of CD45RA (b) and their schematic explanation (d). Amplification will go if a conjugate of antibody against CD3 with duplex **1*4** (green) will be in proximity with a few conjugates of antibody against CD45RA with strand **2** (red). (c) Distribution of acquired Fluorescein (result of cascade reaction, please see Figure S7a) vs CD45RO expression on lymphocyte surfaces after cascade reaction. Distribution presents cells gated on time course between 1300 and 1500 seconds.
